# 2-[Carbamo­thio­yl(2-hy­droxy­eth­yl)amino]­ethyl benzoate: crystal structure, Hirshfeld surface analysis and computational study

**DOI:** 10.1107/S2056989020006829

**Published:** 2020-05-29

**Authors:** Sang Loon Tan, Edward R. T. Tiekink

**Affiliations:** a Research Centre for Crystalline Materials, School of Science and Technology, Sunway University, 47500 Bandar Sunway, Selangor Darul Ehsan, Malaysia

**Keywords:** crystal structure, thio­urea, hydrogen bonding, Hirshfeld surface analysis, computational chemistry

## Abstract

The title di-substituted thio­urea, C_12_H_16_N_2_O_3_S, has the hy­droxy­lethyl and ethyl benzoate substituents bound to the same amine-N atom, and is twisted, having a (+)syn-clinal conformation with the N_amine_—C—C—O_(hydroxyl, carbon­yl)_ torsion angles of 49.39 (13) and 59.09 (12)°, respectively; the dihedral angle between the almost planar CN_2_S core and the pendent benzene ring is 69.26 (4)°. In the crystal, supra­molecular layers propagating in the *ac* plane are formed *via* a combination of hydroxyl-O—H⋯S(thione), amine-N—H⋯O(hydroxyl, carbon­yl) hydrogen-bonds. The layers stack along the *b* axis with inter-digitation of the benzene rings allowing the formation of π–π stacking [inter-centroid separation = 3.8722 (7) Å] and parallel C=O⋯π inter­actions. A computational chemistry study shows the conventional hydrogen bonding in the crystal leads to significant electrostatic stabilization but dispersion terms are also apparent, notably through the inter­actions involving the benzene residue.

## Abstract 2

The title di-substituted thio­urea has hy­droxy­lethyl and ethyl benzoate substituents bound to the same amine-N atom; overall the mol­ecule is twisted. Supra­molecular layers are formed in the crystal, with the mol­ecules connected by O—H⋯S and N—H⋯O(carbonyl, hydrox­yl) hydrogen bonds.

## Chemical context   

The title compound, (I), was characterized crystallographically in a continuation of recent structural studies of tri-substituted thio­urea derivatives formulated as (HOCH_2_CH_2_)_2_NC(=S)N(H)C(=O)C_6_H_4_-*R*-4 for *R* = Me (Tan, Azizan *et al.*, 2019 ▸) and *R* = NO_2_ (Tan *et al.*, 2020 ▸): these mol­ecules are known for their various applications including biological activity (Saeed *et al.*, 2014 ▸). A convenient synthesis for these mol­ecules is *via* the reaction of NH_4_(NCS), *R*_2_NH and *Ar*C(=O)Cl to yield *R*_2_NC(=S)N(H)C(=O)*Ar*. In an experiment with *R* = CH_2_CH_2_OH and *Ar* = C_6_H_5_, the solution was also heated resulting in an apparent rearrangement with deprotonation of one hy­droxy­ethyl group followed by nucleophilic attachment at the carbonyl-C atom along with protonation of the primary amine and cleavage of the original N—C(=O) bond to yield (I), formulated as H_2_NC(=S)N(CH_2_CH_2_OH)CH_2_CH_2_OC(=O)C_6_H_5_. The mol­ecular structure of (I) was determined by X-ray crystallography and the supra­molecular association investigated by Hirshfeld surface analysis and computational chemistry.
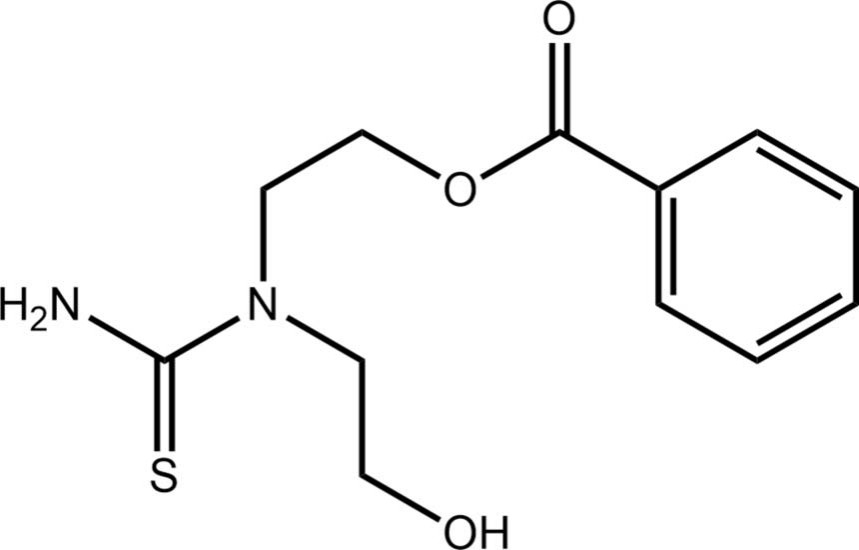


## Structural commentary   

**Figure 1:**
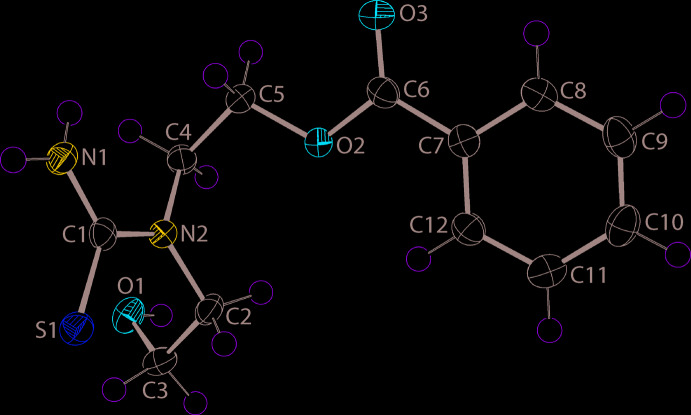
The mol­ecular structure of (I) showing the atom-labelling scheme and displacement ellipsoids at the 70% probability level.

The mol­ecule of (I) is shown in Fig. 1 ▸ and comprises a di-substituted thio­urea mol­ecule with both substitutions occurring at the same amine atom. The CN_2_S atoms of the thio­urea core are almost planar, exhibiting a r.m.s. deviation = 0.0054 Å, with the appended C2 and C4 atoms lying 0.0236 (18) and 0.0216 (16) Å to either side of the plane. The conformation of the C2-hy­droxy­lethyl residue is (+)syn-clinal as indicated by the N2—C2—C3—O1 torsion angle of 49.39 (13)°. The CO_2_ residue is close to co-planar with the (C7–C12)-benzene ring to which it is connected, forming a dihedral angle of 4.83 (9)°. The dihedral angle between the least-squares planes through the CN_2_S core and the benzene ring is 69.26 (4)°, indicating the mol­ecule is highly twisted. Finally, the N2—C4—C5—O2 torsion angle of 59.09 (12)° is indicative of a (+)syn-clinal configuration about the C—C bond, thereby confirming the twisted nature of the mol­ecule.

## Supra­molecular features   

**Figure 2:**
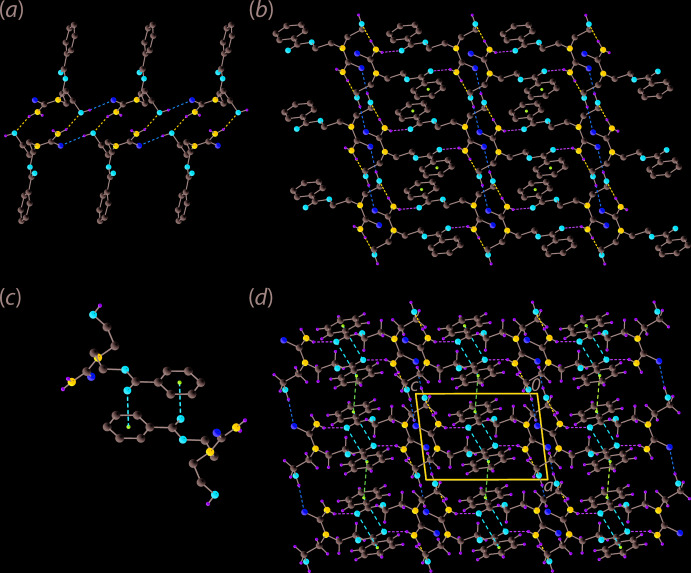
Mol­ecular packing in the crystal of (I): (*a*) supra­molecular tape along the *a* axis mediated by hydroxyl-O—H⋯S(thione) and amine-N—H⋯O(hydrox­yl) hydrogen bonding shown as orange and blue dashed lines, respectively, (*b*) supra­molecular layer where the tapes of (*a*) are connected by amine-N—H⋯O(carbon­yl) hydrogen bonds shown as green dashed lines, (*c*) detail of C—O⋯π(benzene) inter­actions shown as red dashed lines and (*d*) a view of the unit-cell contents down the *b* axis with π(benzene)–π(benzene) inter­actions shown as purple dashed lines.

As anti­cipated, hydrogen bonding plays a key role in the supra­molecular assembly of (I); see Table 1 ▸ for geometrical data. The combination of hydroxyl-O—H⋯S(thione) and amine-N—H⋯O(hydrox­yl) hydrogen bonds connect mol­ecules into a supra­molecular tape propagating along the *a*-axis direction, Fig. 2 ▸(*a*). These hydrogen bonds also lead to the formation of 12-membered {⋯HO⋯HNCS}_2_ and 14-membered {⋯OC_2_NCNH}_2_ synthons, each disposed about a centre of inversion, and linked *via* the edges defined by the amine-N—H⋯O(hydrox­yl) hydrogen bonds. The tape has a step-ladder topology and projecting laterally to either side of the tape are the remaining amine-H and carbonyl-O atoms, which form the donors and acceptors of amine-N—H⋯O(carbon­yl) hydrogen bonds to link the tapes into a layer in the *ac* plane, Fig. 2 ▸(*b*). The directional links between layers are twofold, namely π–π stacking between the centrosymmetrically related benzene rings [inter-centroid separation = 3.8722 (7)° for symmetry operation 2 − *x*, 1 − *y*, 1 − *z*] and parallel C=O⋯π inter­actions, Table 1 ▸ and Fig. 2 ▸(*c*). These inter­actions are possible owing to the inter-digitation of the benzene rings along the *b*-axis direction, as highlighted in Fig. 2 ▸(*d*).

## Hirshfeld surface analysis   

**Figure 3:**
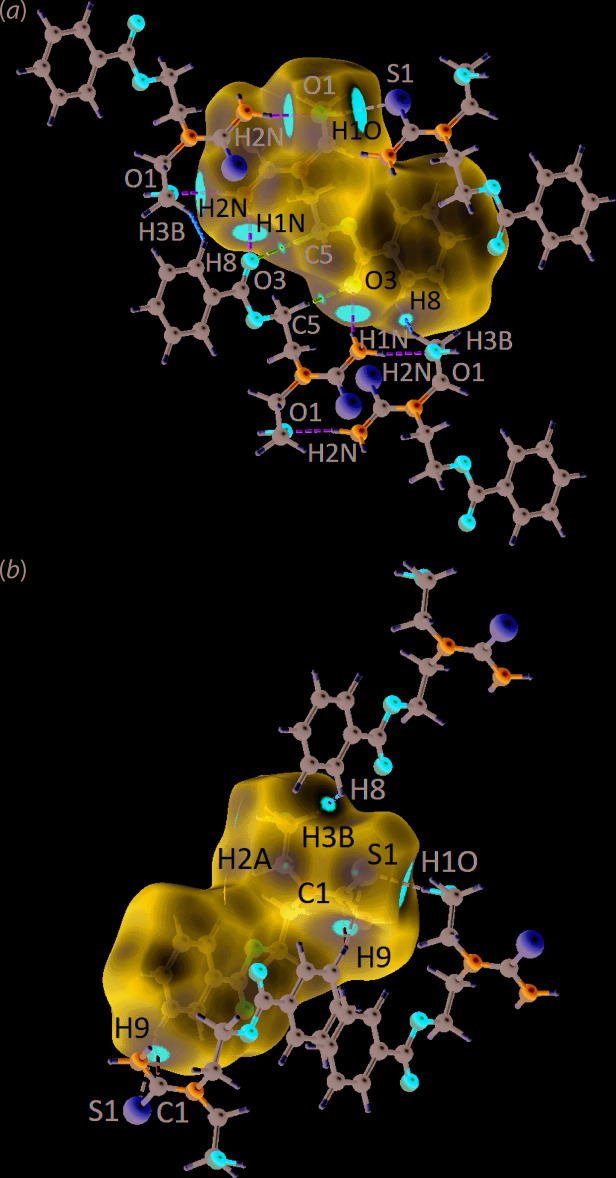
The two views of the *d*_norm_ maps for (I), showing the relevant short contacts indicated by the red spots on the Hirshfeld surface with varying intensities within the range −0.0322 to 1.1699 arbitrary units for (*a*) H1*B*⋯O1, H1*O*⋯S1, H1*A*⋯O3, C5⋯O3 and H3*B*⋯H8 and (*b*) H9⋯C1, H9⋯S1 as well as H2*A*⋯S1 (not connected for clarity). All H⋯O/O⋯H inter­actions are indicated in green, H⋯S/S⋯H in black, H⋯C/C⋯H in light blue, C⋯O/O⋯C in pink and H⋯H in orange.

Structure (I) was subjected to a Hirshfeld surface analysis in order to gain further understanding into the mol­ecular inter­actions existing within the crystal. This was achieved through *Crystal Explorer 17* (Turner *et al.*, 2017 ▸) using established methods (Tan, Jotani *et al.*, 2019 ▸). A list of *d*_norm_ contact distances for all identified inter­actions is given in Table 2 ▸. As noted from Fig. 3 ▸, several red spots of variable intensity were identified on the Hirshfeld surface of (I), being indicative of close inter­actions with contact distances shorter than the sum of the respective van der Waals (vdW) radii (Spackman & Jayatilaka, 2009 ▸). In particular, the most intense red spot is observed for the amine-N1—H2*N*⋯O1(hydrox­yl) hydrogen bond with a *d*_norm_ distance of 1.92 Å, which is significantly shorter, by 0.69 Å [= Δ|(*d*_norm_ – ΣvdW)_H⋯O_| in Table 2 ▸], than the vdW value of 2.61 Å (adjusted to neutron values). Other prominent features are due to the hydroxyl-O1—H1*O*⋯S1(thione) and amine-N1—H1*N*⋯O3(carbon­yl) hydrogen bonds. Less intense features on the *d*_norm_ maps of Fig. 3 ▸ are due to benzene-C9—H9⋯C1(thione) and methyl­ene-C3—H3*B*⋯H8(benzene) inter­actions, and the diminutive spots arise from weaker methyl­ene-C5⋯O3(carbon­yl), methyl­ene-C2—H2*A*⋯S1(thione) and benzene-C9—H9⋯S1(thione) contacts at distances just shorter or approximately equivalent to the values of the respective ΣvdW radii. Apart from the conventional hydrogen bonds and other inter­actions involving hydrogen, several inter­actions involving the aromatic ring are apparent.

**Figure 4:**
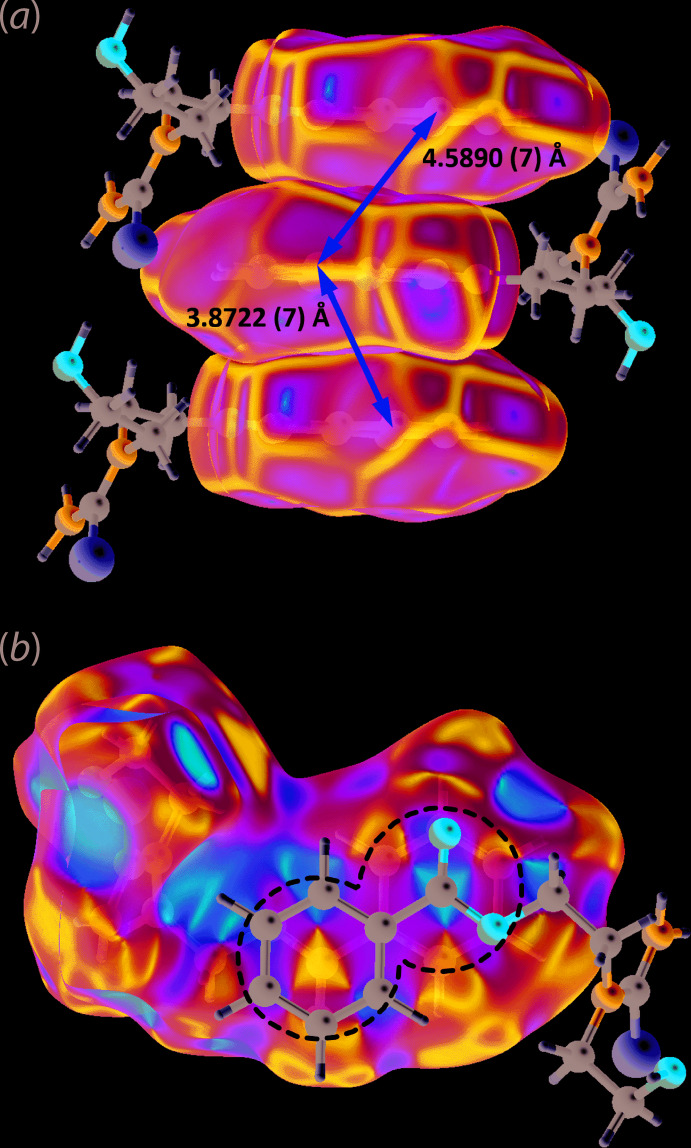
(*a*) The Hirshfeld surface mapped with curvedness (property range: −4.0 to +0.4 arbitrary units) for the benzoate fragments of (I), showing the shape complementarity for the π–π stacking between the fragments and (*b*) the shape-index (property range: −1.0 to +1.0 arbitrary units) on the Hirshfeld surface of (I), showing the concave (red) and convex (blue) regions indicating the C⋯O shape complementary inter­action (circled).

Thus, π(benzene)–π(benzene) inter­actions, with an inter-centroid separation = 3.8722 (7) Å, as well as parallel C6=O3⋯*Cg*(C7–C11) inter­actions, occurring on either side of a reference benzene ring, are validated through further Hirshfeld surface analysis. The presence of π–π inter­actions are supported by the shape complementarity between the aromatic rings as evidenced from the planar stacking arrangement illustrated through the Hirshfeld surface mapped with curvedness in Fig. 4 ▸(*a*). As for the C=O⋯*π* inter­action, the shape-index on the Hirshfeld surface reveals that there are complementary concave and convex shapes indicated by the red and blue regions around the centre of aromatic ring and ester-C6 atom, respectively, in Fig. 4 ▸(*b*). This suggests the inter­action could involve a significant contribution from the C6 atom; the C6⋯*Cg*(benzene) separation is 3.5026 (11) Å as opposed to the O3⋯*Cg*(benzene) separation of 3.6604 (10) Å, Table 1 ▸.

**Figure 5:**
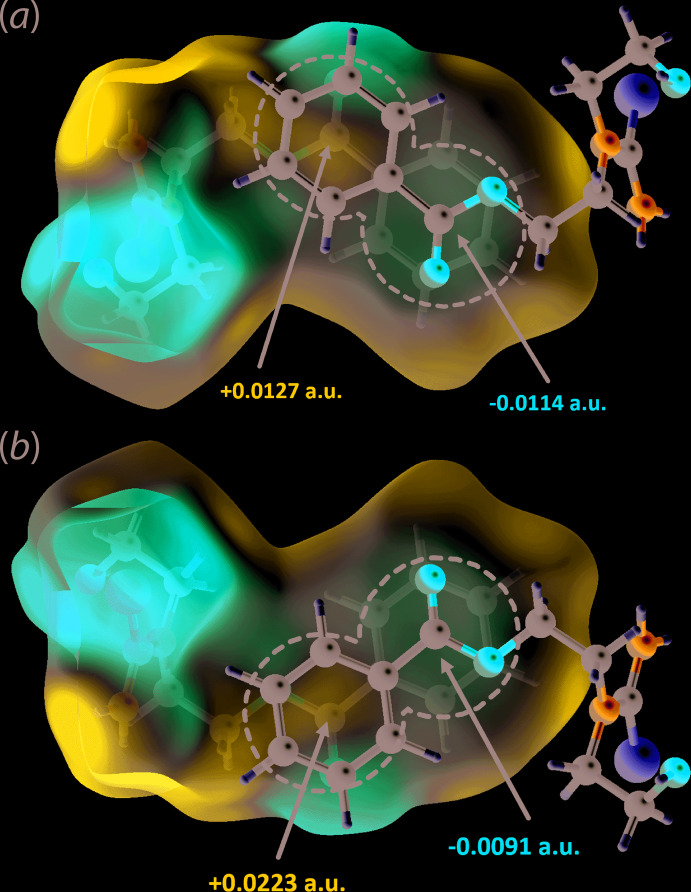
The electrostatic potential mapped onto the Hirshfeld surface for (I) within the range −0.0672 to 0.0620 atomic units for (*a*) the upper side of the ester group (circled blue region) and π-ring system (circled red region) and (*b*) the reverse sides of the ester group (circled faint-blue region) and π-ring system (circled faint-red region). The images highlight the charge complementarity between the specified inter­actions.

In order to confirm the above findings, particularly the short contacts as well as the inter­actions involving the aromatic ring, electrostatic potential (ESP) mapping was also performed on the Hirshfeld surface using the DFT-B3LYP quantum level of theory and 6-31G(*d*,*p*) basis set as available in *Crystal Explorer 17* (Turner *et al.*, 2017 ▸). The ESP charge for each H-atom donor and acceptor of the relevant close contacts are tabulated in Table 3 ▸. As expected for the conventional hydrogen bonds detected through *PLATON* (Spek, 2020 ▸), significant differences are observed in the electrostatic potentials of the hydrogen-bond donor and acceptor atoms, indicating a strong attraction. Similar observations are noted for the other identified contacts but with smaller differences with the notable exception of the methyl­ene-C3—H3*B*⋯H8(benzene) contact, for which both inter­acting hydrogen atoms exhibit a positive electrostatic potential signifying that the inter­action is dispersive in nature. As for the π–π inter­action, it has already been established that the contacts arise to charge complementarity between the rings. Concerning the C=O3⋯π contact, occurring between benzene rings separated by an inter-centroid separation of 4.5890 (7) Å, the ester-C6 atom exhibits positive ESP of +0.0127 a.u. on one side to complement the negative ESP of −0.0114 a.u. at the centre of the aromatic ring it inter­acts with, Fig. 5 ▸(*a*). At the same time it has an ESP charge of +0.0223 a.u. on the reverse side that complements the other side of a symmetry related aromatic ring, involved in the π–π contact with an inter-centroid distance of 3.8722 (7) Å, with the ESP charge of −0.0091 a.u., Fig. 5 ▸(*b*).

**Figure 6:**
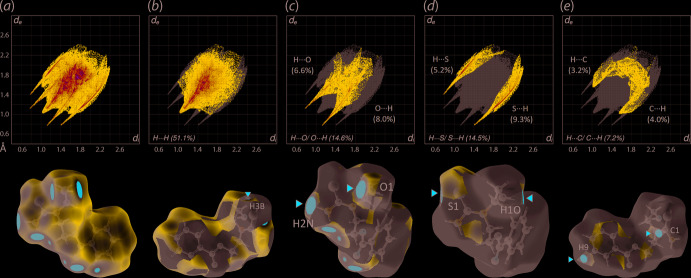
Upper view: (*a*) The overall two-dimensional fingerprint plot for (I) and those delineated into (*b*) H⋯H, (*c*) H⋯O/O⋯H, (*d*) H⋯S/S⋯H and (e) H⋯C/C⋯H, (*e*) contacts, with the percentage contributions to the overall surface specified within each plot. Lower views: *d*_norm_ maps where the tip of the delineated fingerprint plot corresponds to the relevant contact on the Hirshfeld surface and identified through the red cursors.

The close contacts were also investigated through fingerprint plot analysis, shown in the upper views of Fig. 6 ▸. The *d*_norm_-mapped Hirshfeld surfaces for the most prominent point-to-point inter­actions, giving rise to the most discernible peaks in the fingerprint plots, are shown in the lower views of Fig. 6 ▸. In general, (I) exhibits a paw-like, overall fingerprint profile, Fig. 6 ▸(*a*), which can be mainly delineated into H⋯H (51.1%), H⋯O/ O⋯H (14.6%), H⋯S/ S⋯H (14.5%), H⋯C/ C⋯H (7.2%), C⋯C (6.0%) contacts, Fig. 6 ▸(*b*)–(*e*), as well as other minor contacts which constitute about 6.0% of the remaining contacts. A further analysis on the respective fingerprint plots shows that the distribution for the (inter­nal)-O⋯H-(external), (inter­nal)-S⋯H-(external) and (inter­nal)-C⋯H-(external) are slightly more dominant than the (inter­nal)-H⋯*X*-(external) counterparts (*X* = O, S, and C), with the distribution being 8.0, 9.3 and 4.0% as against 6.6, 5.2 and 3.2%, respectively. These results tally with the fact that (I) has more hydrogen-bond acceptors than hydrogen-bond donor atoms. Nonetheless, both (inter­nal)-*X*⋯H-(external) and (inter­nal)-H⋯*X*-(external) exhibit equivalent contact distances that are tipped at the minimum *d*_i_ + *d*_e_ values, which correspond to the specified contacts in Table 2 ▸.

## Computational chemistry   

The calculation of the inter­action energy for all pairwise mol­ecules in (I) was performed through *Crystal Explorer 17* (Turner *et al.*, 2017 ▸) with the purpose of studying the strength of each inter­action/set of inter­actions identified from the Hirshfeld surface analysis. Hence, the electrostatic (*E*_ele_), polarization (*E*_pol_), dispersion (*E*_dis_) and exchange-repulsion (*E*_rep_) terms were calculated with the results tabulated in Table 4 ▸.

Among all the inter­actions, it is the amine-N1—H2*N*⋯O1(hydrox­yl) hydrogen bond, that closes the connected 12-membered {⋯HO⋯HNCS}_2_ and 14-membered {⋯OC_2_NCNH}_2_ synthons, that has the greatest inter­action energy, *E*_int_ = −85.6 kJ mol^−1^. Next most stabil­izing are the amine-N1—H1*N*⋯O3(carbon­yl) and methyl­ene-C5⋯O3(carbon­yl) contacts between centrosym­metrically related mol­ecules [−66.1 kJ mol^−1^], the ester-C6⋯π(benzene), benzene-C9—H9⋯S1(thione) and benzene-C9–H9⋯C1(thione) contacts with a combined *E*_int_ of −48.3 kJ mol^−1^ and hydroxyl-O1–H1*O*⋯S1(thione) [−28.8 kJ mol^−1^]. Close in energy to latter is that due to π–π [*Cg*1⋯*Cg*1 = 3.8722 (7) Å] with *E*_int_ = −28.3 kJ mol^−1^. Next most significant are the pairwise ethyl­ene-C2—H2*A*⋯S1(thione) inter­actions (*E*_int_ = −23.1 kJ mol^−1^) then methyl­ene-C3—H3*B*⋯H8(benzene) (*E*_int_ = −4.3 kJ mol^−1^).

**Figure 7:**
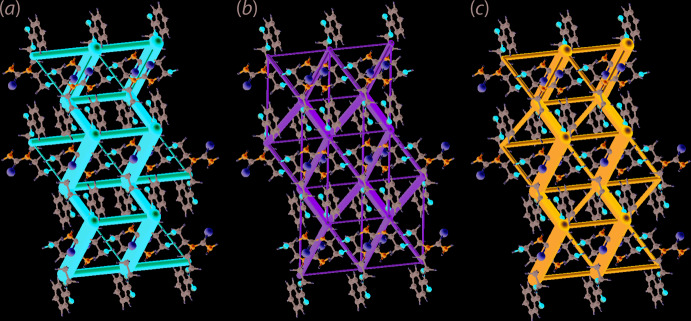
Perspective views of the energy frameworks of (I), showing the (*a*) electrostatic force, (*b*) dispersion force and (*c*) total energy. The radii of the cylinders are proportional to the relative strength of the corresponding energies and were adjusted to the same scale factor of 100 with a cut-off value of 8 kJ mol^−1^ within a 2 × 2 × 2 unit cells.

The crystal of (I) is mainly sustained by electrostatic forces owing to the presence of the relatively strong hydrogen-bonding inter­actions, *viz*. amine-N1—H1*N*⋯O3(carbon­yl) that propagates along the *c* axis together with amine-N1—H2*N*⋯O1(hydrox­yl) and hydroxyl-O1—H1*O*⋯S1(thione), which extend along the *a* axis, thereby forming a step-ladder framework as shown in Fig. 7 ▸(*a*). On the other hand, significant dispersion force is also present as evidenced from the wire mesh-like dispersion energy framework predominantly governed by the *π*–*π* inter­actions, with contributions from the inter­actions involving the benzene-C9 atom, Fig. 7 ▸(*b*). Overall, the combination of electrostatic and dispersion forces leads to a cuboid-like framework shown in Fig. 7 ▸(*c*).

## Database survey   

**Figure 8:**
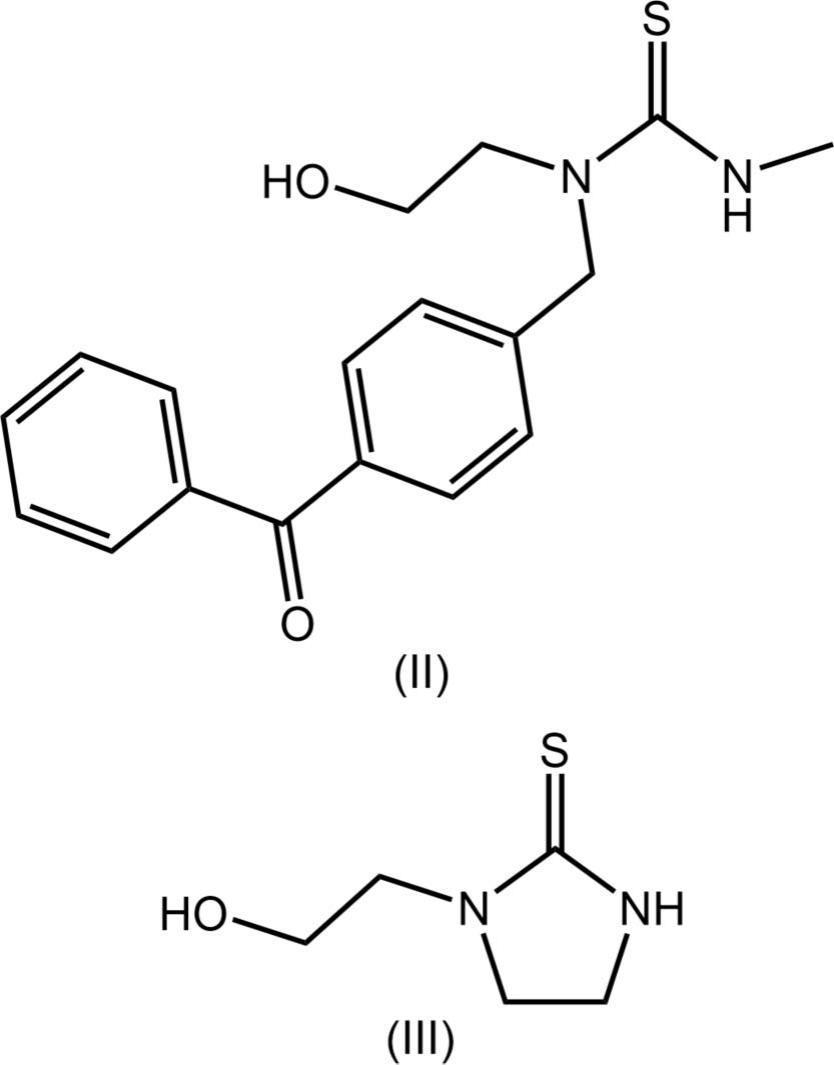
Chemical diagrams for (II) and (III).

Crystal-structure determinations of organic mol­ecules of the general formula *R*(*R*′)NC(=S)NH_2_ are comparatively rare with the simplest derivative being the *R* = *R*′ = Me species, the almost planar mol­ecule being first reported in 1994 (WIFKOL; Pathirana *et al.*, 1994 ▸). Similarly, derivatives bearing hydroxyl groups are uncommon and include the relatively simple derivatives shown in Fig. 8 ▸, *i.e*. acyclic (II) (IYAYAJ; Griffiths *et al.*, 2010 ▸) and cyclic imidazolidine-2-thione (III) (DOJSUT; Lee *et al.*, 2018 ▸).

## Synthesis and crystallization   

Compound (I) was synthesized by gently heating an acetone mixture (30 ml) containing ammonium thio­cyanate (Fisher, 1 mmol), benzoyl chloride (Acros, 1 mmol) and bis­(hy­droxy­eth­yl)amine (Acros, 1 mmol). The solution was concentrated to half of the initial volume under heating and a white precipitate was obtained upon cooling the solution to room temperature. Colourless blocks were formed through recrystallization of the crude product from acetone solution. M.p. 388.6–390.1 K. IR (cm^−1^): 3419 *ν*(OH), 3323 *ν*(NH_2_)_asym_, 3222 *ν*(NH_2_)_sym_, 3058 *ν*(CH)_arom_, 3002–2881 *ν*(CH), 1706 *ν*(COO), 1647 *ν*(C=O), 1600 *δ*(NH), 1523 *ν*(C=C), 1270 *ν*(CN), 1053 *ν*(C=S), 711 *δ*(CH).

## Refinement   

Crystal data, data collection and structure refinement details are summarized in Table 5 ▸. The carbon-bound H atoms were placed in calculated positions (C—H = 0.95–0.98 Å) and were included in the refinement in the riding-model approximation, with *U*_iso_(H) set to 1.2*U*_eq_(C). The oxygen- and nitro­gen-bound H atoms were located from a difference-Fourier map and refined with O—H = 0.84±0.01 Å and N—H = 0.88±0.01 Å, and with *U*_iso_(H) set to 1.5*U*_eq_(O) or 1.2*U*_eq_(N).

## Supplementary Material

Crystal structure: contains datablock(s) I, global. DOI: 10.1107/S2056989020006829/hb7918sup1.cif

Structure factors: contains datablock(s) I. DOI: 10.1107/S2056989020006829/hb7918Isup2.hkl

Supporting information file. DOI: 10.1107/S2056989020006829/hb7918Isup3.cml

CCDC reference: 2004940

Additional supporting information:  crystallographic information; 3D view; checkCIF report

## Figures and Tables

**Table 1:** Hydrogen-bond geometry (Å, °) *Cg*1 is the centroid of the (C7–C12) ring.

*D*—H⋯*A*	*D*—H	H⋯*A*	*D*⋯*A*	*D*—H⋯*A*
O1—H1*O*⋯S1^i^	0.84 (1)	2.35 (1)	3.1746 (9)	169 (2)
N1—H2*N*⋯O1^ii^	0.88 (1)	2.04 (1)	2.8582 (13)	155 (1)
N1—H1*N*⋯O3^iii^	0.88 (1)	2.30 (1)	3.1218 (13)	158 (1)
C6—O3⋯*Cg*1^iv^	1.21 (1)	3.66 (1)	3.5026 (12)	73 (1)

Symmetry codes: (i) 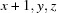; (ii) 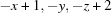; (iii) 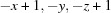; (iv) 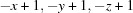.

**Table 2:** A summary of short inter­atomic contacts (Å) for (I)^*a*^

Contact	Distance	ΣvdW	Δ|(*d*_norm_ −ΣvdW)|	Symmetry operation
H2*N*⋯O1^*b*^	1.92	2.61	0.69	1 − *x*, −*y*, 2 − *z*
H1*O*⋯S1^*b*^	2.21	2.89	0.68	1 + *x*, *y*, *z*
H1*N*⋯O3^*b*^	2.17	2.61	0.44	1 − *x*, −*y*, 1 − *z*
H3*B*⋯H8	2.08	2.18	0.10	*x*, *y*, 1 + *z*
H9⋯C1	2.60	2.79	0.19	1 − *x*, 1 − *y*, 1 − *z*
C5⋯O3	3.17	3.22	0.05	1 − *x*, − *y*, 1 − *z*
H2*A*⋯S1	2.87	2.89	0.02	1 − *x*, 1 − *y*, 2 − *z*
H9⋯S1	2.89	2.89	0.00	1 − *x*, 1 − *y*, 1 − *z*

Notes: (*a*) The inter­atomic distances are calculated in *Crystal Explorer 17* (Turner *et al.*, 2017 ▸) whereby the *X*—H bond lengths are adjusted to their neutron values; (*b*) these inter­actions correspond to conventional hydrogen bonds.

**Table 3:** Electrostatic potential charge (*V*_ESP_) for each hydrogen atom donor and acceptor in (I) participating in a close contact identified through Hirshfeld surface analysis

Contact	Electrostatic potential, *V*_ESP_ (a.u.)		Δ|*V*_ESP_|
	H-donor	H-acceptor	
H2*N*⋯O1	0.1446	−0.0654	0.2100
H1*O*⋯S1	0.1488	−0.0607	0.2095
H1*N*⋯O3	0.1248	−0.0601	0.1849
H9⋯C1	0.0441	−0.0119	0.0560
H3*B*⋯H8	0.0066	0.0229	0.0163
C5⋯O3	0.0581	−0.0562	0.1143
H2*A*⋯S1	0.0239	−0.0589	0.0828
H9⋯S1	0.0219	−0.0458	0.0677

**Table 4:** A summary of inter­action energies (kJ mol^−1^) calculated for (I)

Contact	*E*_ele_	*E*_pol_	*E*_dis_	*E*_rep_	*E*_tot_	Symmetry operation
{N1—H2*N*⋯O1}_2_	−91.6	−13.5	−39.5	59.1	−85.6	1 − *x*, − *y*, 2 − *z*
{N1—H1*N*⋯O3}_2_ + {C5⋯O3}_2_	−56.9	−10.1	−26.5	27.4	−66.1	1 − *x*, − *y*, 1 − *z*
C6⋯π(benzene) +						
{C9—H9⋯S1}_2_ +						
{C9—H9⋯C1}_2_	−21.6	−3.0	−57.1	33.2	−48.3	1 − *x*, 1 − *y*, 1 − *z*
O1—H1*O*⋯S1	−47.2	−7.5	−10.6	36.5	−28.8	1 + *x*, *y*, *z*
π(benzene)–π(benzene)	−0.4	−1.6	−43.2	17.0	−28.3	2 − *x*, 1 − *y*, 1 − *z*
{C2—H2*A*⋯S1}_2_	−14.6	−5.1	−14.7	11.3	−23.1	1 − *x*, 1 − *y*, 2 − *z*
C3—H3*B*⋯H8	1.1	−1.8	−12.7	9.1	−4.3	*x*, *y*, 1 + *z*

**Table 5:** Experimental details

Crystal data	
Chemical formula	C_12_H_16_N_2_O_3_S
*M*_r_	268.33
Crystal system, space group	Triclinic, *P* 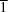
Temperature (K)	100
*a*, *b*, *c* (Å)	7.1608 (2), 8.8771 (2), 10.0728 (2)
α, β, γ (°)	96.815 (2), 96.057 (2), 95.990 (2)
*V* (Å^3^)	627.78 (3)
*Z*	2
Radiation type	Cu *K*α
μ (mm^−1^)	2.33
Crystal size (mm)	0.14 × 0.10 × 0.09

Data collection	
Diffractometer	XtaLAB Synergy, Dualflex, AtlasS2
Absorption correction	Gaussian (*CrysAlis PRO*; Rigaku OD, 2018 ▸)
*T*_min_, *T*_max_	0.656, 1.000
No. of measured, independent and observed [*I* > 2σ(*I*)] reflections	15863, 2608, 2530
*R*_int_	0.028
(sin θ/λ)_max_ (Å^−1^)	0.631

Refinement	
*R*[*F* ^2^ > 2σ(*F* ^2^)], *wR*(*F* ^2^), *S*	0.027, 0.072, 1.02
No. of reflections	2608
No. of parameters	172
No. of restraints	3
H-atom treatment	H atoms treated by a mixture of independent and constrained refinement
Δρ_max_, Δρ_min_ (e Å^−3^)	0.28, −0.29

Computer programs: *CrysAlis PRO* (Rigaku OD, 2018 ▸), *SHELXS* (Sheldrick, 2015*a* ▸), *SHELXL2018/3* (Sheldrick, 2015*b* ▸), *ORTEP-3 for Windows* (Farrugia, 2012 ▸), *DIAMOND* (Brandenburg, 2006 ▸) and*publCIF* (Westrip, 2010 ▸).

## supplementary crystallographic information

### Crystal data

**Table:**

C_12_H_16_N_2_O_3_S	*Z* = 2
*M**_r_* = 268.33	*F*(000) = 284
Triclinic, *P*1	*D*_x_ = 1.420 Mg m^−^^3^
*a* = 7.1608 (2) Å	Cu *K*α radiation, λ = 1.54184 Å
*b* = 8.8771 (2) Å	Cell parameters from 11731 reflections
*c* = 10.0728 (2) Å	θ = 5.0–76.3°
α = 96.815 (2)°	µ = 2.33 mm^−^^1^
β = 96.057 (2)°	*T* = 100 K
γ = 95.990 (2)°	Block, colourless
*V* = 627.78 (3) Å^3^	0.14 × 0.10 × 0.09 mm

### Data collection

**Table:**

XtaLAB Synergy, Dualflex, AtlasS2 diffractometer	2608 independent reflections
Radiation source: micro-focus sealed X-ray tube, PhotonJet (Cu) X-ray Source	2530 reflections with *I* > 2σ(*I*)
Mirror monochromator	*R*_int_ = 0.028
Detector resolution: 5.2558 pixels mm^-1^	θ_max_ = 76.6°, θ_min_ = 4.5°
ω scans	*h* = −8→9
Absorption correction: gaussian (CrysAlisPro; Rigaku OD, 2018)	*k* = −11→11
*T*_min_ = 0.656, *T*_max_ = 1.000	*l* = −12→11
15863 measured reflections	

### Refinement

**Table:**

Refinement on *F*^2^	Primary atom site location: structure-invariant direct methods
Least-squares matrix: full	Secondary atom site location: difference Fourier map
*R*[*F*^2^ > 2σ(*F*^2^)] = 0.027	Hydrogen site location: mixed
*wR*(*F*^2^) = 0.072	H atoms treated by a mixture of independent and constrained refinement
*S* = 1.02	*w* = 1/[σ^2^(*F*_o_^2^) + (0.0387*P*)^2^ + 0.2837*P*] where *P* = (*F*_o_^2^ + 2*F*_c_^2^)/3
2608 reflections	(Δ/σ)_max_ = 0.001
172 parameters	Δρ_max_ = 0.28 e Å^−^^3^
3 restraints	Δρ_min_ = −0.29 e Å^−^^3^

### Special details

**Table:**

Geometry. All esds (except the esd in the dihedral angle between two l.s. planes) are estimated using the full covariance matrix. The cell esds are taken into account individually in the estimation of esds in distances, angles and torsion angles; correlations between esds in cell parameters are only used when they are defined by crystal symmetry. An approximate (isotropic) treatment of cell esds is used for estimating esds involving l.s. planes.

### Fractional atomic coordinates and isotropic or equivalent isotropic displacement parameters (Å^2^)

**Table:**

	*x*	*y*	*z*	*U*_iso_*/*U*_eq_	
S1	0.37572 (4)	0.27008 (3)	1.03290 (3)	0.01701 (9)	
O1	0.95223 (11)	0.15258 (9)	1.06895 (9)	0.02017 (19)	
H1O	1.0641 (15)	0.1717 (19)	1.0521 (17)	0.030*	
O2	0.70466 (11)	0.26850 (9)	0.61989 (8)	0.01658 (17)	
O3	0.60618 (13)	0.20970 (10)	0.39867 (8)	0.02231 (19)	
N1	0.35269 (14)	0.03143 (11)	0.84534 (10)	0.0167 (2)	
H1N	0.396 (2)	−0.0360 (14)	0.7896 (13)	0.020*	
H2N	0.2480 (16)	0.0025 (16)	0.8779 (14)	0.020*	
N2	0.63866 (13)	0.18528 (10)	0.87909 (9)	0.01420 (19)	
C1	0.46322 (16)	0.15473 (12)	0.91223 (11)	0.0146 (2)	
C2	0.76658 (16)	0.31983 (13)	0.94513 (12)	0.0169 (2)	
H2A	0.692935	0.407323	0.960760	0.020*	
H2B	0.861614	0.347207	0.884739	0.020*	
C3	0.86760 (16)	0.29131 (13)	1.07870 (12)	0.0183 (2)	
H3A	0.966872	0.377706	1.111775	0.022*	
H3B	0.775824	0.287765	1.145528	0.022*	
C4	0.71451 (16)	0.08514 (13)	0.77635 (11)	0.0158 (2)	
H4A	0.676376	−0.022672	0.787412	0.019*	
H4B	0.854379	0.103147	0.790497	0.019*	
C5	0.64657 (16)	0.11088 (13)	0.63421 (11)	0.0170 (2)	
H5A	0.702102	0.042030	0.568493	0.020*	
H5B	0.507114	0.088915	0.617061	0.020*	
C6	0.67210 (15)	0.30367 (13)	0.49377 (11)	0.0162 (2)	
C7	0.72974 (15)	0.46730 (13)	0.48446 (11)	0.0158 (2)	
C8	0.70318 (16)	0.51705 (14)	0.35804 (12)	0.0180 (2)	
H8	0.641338	0.448875	0.282694	0.022*	
C9	0.76728 (17)	0.66610 (14)	0.34284 (12)	0.0214 (2)	
H9	0.752018	0.699503	0.256588	0.026*	
C10	0.85375 (17)	0.76653 (14)	0.45357 (13)	0.0218 (3)	
H10	0.899045	0.868220	0.442592	0.026*	
C11	0.87453 (17)	0.71919 (14)	0.58064 (13)	0.0212 (2)	
H11	0.930722	0.789112	0.656580	0.025*	
C12	0.81286 (16)	0.56950 (13)	0.59597 (12)	0.0182 (2)	
H12	0.827287	0.536650	0.682476	0.022*	

### Atomic displacement parameters (Å^2^)

**Table:**

	*U*^11^	*U*^22^	*U*^33^	*U*^12^	*U*^13^	*U*^23^
S1	0.01619 (15)	0.01550 (15)	0.01948 (15)	0.00207 (10)	0.00422 (10)	0.00084 (10)
O1	0.0143 (4)	0.0183 (4)	0.0283 (5)	0.0016 (3)	0.0032 (3)	0.0041 (3)
O2	0.0206 (4)	0.0148 (4)	0.0137 (4)	−0.0014 (3)	0.0016 (3)	0.0025 (3)
O3	0.0282 (5)	0.0199 (4)	0.0162 (4)	−0.0027 (3)	−0.0008 (3)	−0.0003 (3)
N1	0.0162 (5)	0.0147 (5)	0.0184 (5)	−0.0008 (4)	0.0025 (4)	0.0015 (4)
N2	0.0149 (4)	0.0131 (4)	0.0141 (4)	0.0001 (3)	0.0018 (3)	0.0012 (3)
C1	0.0162 (5)	0.0140 (5)	0.0144 (5)	0.0020 (4)	0.0001 (4)	0.0057 (4)
C2	0.0170 (5)	0.0127 (5)	0.0203 (5)	−0.0024 (4)	0.0030 (4)	0.0017 (4)
C3	0.0170 (5)	0.0157 (5)	0.0203 (6)	−0.0002 (4)	0.0005 (4)	−0.0021 (4)
C4	0.0172 (5)	0.0146 (5)	0.0160 (5)	0.0027 (4)	0.0033 (4)	0.0019 (4)
C5	0.0203 (5)	0.0136 (5)	0.0163 (5)	−0.0011 (4)	0.0030 (4)	0.0007 (4)
C6	0.0139 (5)	0.0201 (6)	0.0147 (5)	0.0019 (4)	0.0024 (4)	0.0019 (4)
C7	0.0134 (5)	0.0176 (6)	0.0169 (5)	0.0030 (4)	0.0029 (4)	0.0028 (4)
C8	0.0178 (5)	0.0207 (6)	0.0160 (5)	0.0057 (4)	0.0015 (4)	0.0017 (4)
C9	0.0234 (6)	0.0233 (6)	0.0206 (6)	0.0095 (5)	0.0048 (5)	0.0085 (5)
C10	0.0209 (6)	0.0164 (5)	0.0301 (6)	0.0050 (4)	0.0058 (5)	0.0061 (5)
C11	0.0202 (6)	0.0190 (6)	0.0231 (6)	0.0024 (4)	0.0001 (5)	−0.0007 (5)
C12	0.0180 (5)	0.0204 (6)	0.0162 (5)	0.0027 (4)	0.0009 (4)	0.0028 (4)

### Geometric parameters (Å, º)

**Table:**

S1—C1	1.7082 (11)	C4—C5	1.5141 (15)
O1—C3	1.4259 (14)	C4—H4A	0.9900
O1—H1O	0.840 (9)	C4—H4B	0.9900
O2—C6	1.3462 (14)	C5—H5A	0.9900
O2—C5	1.4460 (13)	C5—H5B	0.9900
O3—C6	1.2121 (14)	C6—C7	1.4847 (16)
N1—C1	1.3454 (15)	C7—C12	1.3941 (16)
N1—H1N	0.874 (9)	C7—C8	1.3974 (16)
N1—H2N	0.876 (9)	C8—C9	1.3859 (17)
N2—C1	1.3424 (15)	C8—H8	0.9500
N2—C2	1.4706 (14)	C9—C10	1.3876 (18)
N2—C4	1.4688 (14)	C9—H9	0.9500
C2—C3	1.5203 (16)	C10—C11	1.3919 (18)
C2—H2A	0.9900	C10—H10	0.9500
C2—H2B	0.9900	C11—C12	1.3876 (17)
C3—H3A	0.9900	C11—H11	0.9500
C3—H3B	0.9900	C12—H12	0.9500
			
C3—O1—H1O	108.6 (12)	H4A—C4—H4B	107.8
C6—O2—C5	114.66 (8)	O2—C5—C4	108.15 (9)
C1—N1—H1N	122.3 (10)	O2—C5—H5A	110.1
C1—N1—H2N	117.4 (10)	C4—C5—H5A	110.1
H1N—N1—H2N	117.2 (14)	O2—C5—H5B	110.1
C1—N2—C2	121.80 (9)	C4—C5—H5B	110.1
C1—N2—C4	121.91 (9)	H5A—C5—H5B	108.4
C2—N2—C4	116.29 (9)	O3—C6—O2	122.83 (10)
N2—C1—N1	118.46 (10)	O3—C6—C7	124.38 (10)
N2—C1—S1	121.95 (8)	O2—C6—C7	112.78 (9)
N1—C1—S1	119.57 (9)	C12—C7—C8	119.92 (11)
N2—C2—C3	112.00 (9)	C12—C7—C6	122.18 (10)
N2—C2—H2A	109.2	C8—C7—C6	117.88 (10)
C3—C2—H2A	109.2	C9—C8—C7	119.84 (11)
N2—C2—H2B	109.2	C9—C8—H8	120.1
C3—C2—H2B	109.2	C7—C8—H8	120.1
H2A—C2—H2B	107.9	C8—C9—C10	120.01 (11)
O1—C3—C2	112.86 (9)	C8—C9—H9	120.0
O1—C3—H3A	109.0	C10—C9—H9	120.0
C2—C3—H3A	109.0	C11—C10—C9	120.41 (11)
O1—C3—H3B	109.0	C11—C10—H10	119.8
C2—C3—H3B	109.0	C9—C10—H10	119.8
H3A—C3—H3B	107.8	C12—C11—C10	119.75 (11)
N2—C4—C5	112.81 (9)	C12—C11—H11	120.1
N2—C4—H4A	109.0	C10—C11—H11	120.1
C5—C4—H4A	109.0	C11—C12—C7	120.01 (11)
N2—C4—H4B	109.0	C11—C12—H12	120.0
C5—C4—H4B	109.0	C7—C12—H12	120.0
			
C2—N2—C1—N1	178.51 (9)	O3—C6—C7—C12	−177.03 (11)
C4—N2—C1—N1	−2.38 (15)	O2—C6—C7—C12	1.55 (15)
C2—N2—C1—S1	0.22 (15)	O3—C6—C7—C8	1.20 (17)
C4—N2—C1—S1	179.33 (8)	O2—C6—C7—C8	179.78 (9)
C1—N2—C2—C3	82.67 (13)	C12—C7—C8—C9	2.89 (17)
C4—N2—C2—C3	−96.49 (11)	C6—C7—C8—C9	−175.38 (10)
N2—C2—C3—O1	49.39 (13)	C7—C8—C9—C10	−1.46 (17)
C1—N2—C4—C5	80.00 (13)	C8—C9—C10—C11	−0.84 (18)
C2—N2—C4—C5	−100.85 (11)	C9—C10—C11—C12	1.71 (18)
C6—O2—C5—C4	172.96 (9)	C10—C11—C12—C7	−0.26 (18)
N2—C4—C5—O2	59.09 (12)	C8—C7—C12—C11	−2.03 (17)
C5—O2—C6—O3	−2.74 (15)	C6—C7—C12—C11	176.17 (10)
C5—O2—C6—C7	178.64 (9)		

### Hydrogen-bond geometry (Å, º)

*Cg*1 is the centroid of the (C7–C12) ring.

**Table:**

*D*—H···*A*	*D*—H	H···*A*	*D*···*A*	*D*—H···*A*
O1—H1*O*···S1^i^	0.84 (1)	2.35 (1)	3.1746 (9)	169 (2)
N1—H2*N*···O1^ii^	0.88 (1)	2.04 (1)	2.8582 (13)	155 (1)
N1—H1*N*···O3^iii^	0.88 (1)	2.30 (1)	3.1218 (13)	158 (1)
C6—O3···*Cg*1^iv^	1.21 (1)	3.66 (1)	3.5026 (12)	73 (1)

Symmetry codes: (i) *x*+1, *y*, *z*; (ii) −*x*+1, −*y*, −*z*+2; (iii) −*x*+1, −*y*, −*z*+1; (iv) −*x*+1, −*y*+1, −*z*+1.

## References

[bb1] Brandenburg, K. (2006). *DIAMOND*. Crystal Impact GbR, Bonn, Germany.

[bb2] Farrugia, L. J. (2012). *J. Appl. Cryst.* **45**, 849–854.

[bb3] Griffiths, J.-P., Maliha, B., Moloney, M. G., Thompson, A. L. & Hussain, I. (2010). *Langmuir*, **26**, 14142–14153. 10.1021/la1023482 20672850

[bb4] Lee, S. M., Azizan, A. H. S. & Tiekink, E. R. T. (2018). *Molbank 2018*, article No. M1035.

[bb5] Pathirana, H. M. K. K., Weiss, T. J., Reibenspies, J. H., Zingaro, R. A. & Meyers, E. A. (1994). *Z. Kristallogr. Cryst. Mater* **209**, 698.

[bb6] Rigaku OD (2018). *CrysAlis PRO* Software system. Rigaku Corporation, Oxford, UK.

[bb7] Saeed, A., Flörke, U. & Erben, M. F. (2014). *J. Sulfur Chem.* **35**, 318–355.

[bb8] Sheldrick, G. M. (2015*a*). *Acta Cryst.* A**71**, 3–8.

[bb9] Sheldrick, G. M. (2015*b*). *Acta Cryst.* C**71**, 3–8.

[bb10] Spackman, M. A. & Jayatilaka, D. (2009). *CrystEngComm*, **11**, 19–32.

[bb11] Spek, A. L. (2020). *Acta Cryst.* E**76**, 1–11. 10.1107/S2056989019016244 PMC6944088 31921444

[bb12] Tan, S. L., Azizan, A. H. S., Jotani, M. M. & Tiekink, E. R. T. (2019). *Acta Cryst.* E**75**, 1472–1478. 10.1107/S2056989019012581 PMC6775753 31636978

[bb13] Tan, S. L., Jotani, M. M. & Tiekink, E. R. T. (2019). *Acta Cryst.* E**75**, 308–318. 10.1107/S2056989019001129 PMC6399703 30867939

[bb14] Tan, S. L., Jotani, M. M. & Tiekink, E. R. T. (2020). *Acta Cryst.* E**76**, 155–161. 10.1107/S2056989019017328 PMC7001817 32071739

[bb15] Turner, M. J., Mckinnon, J. J., Wolff, S. K., Grimwood, D. J., Spackman, P. R., Jayatilaka, D. & Spackman, M. A. (2017). *Crystal Explorer 17*. The University of Western Australia.

[bb16] Westrip, S. P. (2010). *J. Appl. Cryst.* **43**, 920–925.

